# Editorial: Human Tumor-Derived p53 Mutants: A Growing Family of Oncoproteins

**DOI:** 10.3389/fonc.2016.00170

**Published:** 2016-07-12

**Authors:** Ygal Haupt, Giovanni Blandino

**Affiliations:** ^1^Tumour Suppression Laboratory, Peter MacCallum Cancer Centre, Melbourne, VIC, Australia; ^2^Sir Peter MacCallum Department of Oncology, The University of Melbourne, Parkville, VIC, Australia; ^3^Sir Peter MacCallum Department of Pathology, The University of Melbourne, Parkville, VIC, Australia; ^4^Department of Biochemistry and Molecular Biology, Monash University, Clayton, VIC, Australia; ^5^Oncogenomic and Epigenetic Unit, Italian National Cancer Institute Regina Elena, Rome, Italy; ^6^Department of Oncology, McMaster University, Hamilton, ON, Canada

**Keywords:** mRNA, p53, gain-of-function, microRNAs, cancers

**The Editorial on the Research Topic**

**Human Tumor-Derived p53 Mutants: A Growing Family of Oncoproteins**

Mutations in the tumor suppressor *p53* gene are collectively the most common event in human cancers. These do not merely reflect a loss of the tumor suppressive function of wild type (wt) p53 but are also selected during tumorigenesis for their acquired gain-of-function (GOF), together contributing to multiple hallmarks of cancer. Over 30 years of extensive study into wt p53 provided a wealth of information about its regulation, functions, and contribution to cancer prevention. Albeit, with a significant delay, the interest in mutant p53 has been growing fast over the past decade with the realization that most cancer patients present tumors with mutant p53, and these particularly manifest in aggressive and metastatic diseases. The growing understanding of mutant p53 exposes attractive therapeutic opportunities with wide clinical applications, which coincidently raise many challenging questions concerning associated complexities. In this research topic, we assembled 12 reviews exposing some critical issues and discussing prospect development in this field.

One of the most commonly used mouse models for cancer is the p53 knockout mouse. However, this model of p53 deficiency does not represent the majority of human cancers. A major leap in the understanding of mutant p53 regulation and GOF was derived from mouse genetics (1, 2). The group of Lozano, which led the mouse models for mutant p53, highlighted the GOF learned from the comparison between p53 deficient mice and mutant p53 knock-in mice, primarily the contribution of mutant p53 to tumor metastasis (Kim et al.). The Lozano group also emphasized the biological and biochemical differences between different mutants, even between different substitutions of the same amino acid, such as p53R172H versus p53R172P. Hence, not all p53 mutants are equal. While the abovementioned mouse models for inherited p53 mutations (the Li–Fraumeni model), this represents a small fraction of p53 mutations in human cancers. This key point was discussed by the Lozano group highlighting the limitations of the current mouse models for sporadic p53 mutations in cancer. They discuss the problems with the current conditional mutant p53 models in which all cells are heterozygous for p53 from conception and hence do not faithfully mimic the role of mutant p53 in sporadic tumor development. These models lack the challenging context of the cells with wt p53 that normally comprise the tumor microenvironment and its inherent immune cells. There is a clear need for more sophisticated mouse models to better define the distinct roles of mutant p53 in these compartments. The Del Sal group (Walerych et al.) discussed the difference between the mouse and human mutant p53 exemplified by the p53R249S mutation, which exhibits GOF in human cells, but this had not been recapitulated in the relevant mouse model. A comprehensive list summarizing studies from the last decade is provided in the Del Sal review outlining which mutants GOF effects have been validated and the associated information.

Mutant p53 GOF requires the accumulation of the mutant protein and that, at least initially, it acts dominantly over the wt protein. Sabapathy discussed this important point in detail, emphasizing the timing and conditions under which dominant negative (DN) effects of mutant p53 occur, and when and how this would impact on tumorigenesis. His conclusion from the literature is that stress, whether acute (e.g., genotoxic stress) or chronic (activated oncogene), accumulates mutant p53; however, it is under the latter conditions that mutant p53 promotes tumorigenesis. Further complexity to this is the tissue specificity of the DN effect as learned from the KI heterozygote mouse models (Sabapathy). In the wake of the DN effect, there is often a loss of heterozygosity (LOH) of the wt allele. However, Walerych et al. discussed that LOH is also tissue specific, as exemplified by the work of Rotter and coworkers demonstrating that, in the embryonic stem cells of the KI mutant p53 mice, the LOH can be of either wt or mutant p53 alleles, potentially acting to control cell fate checkpoint (3). This reflects the opposing effects of wt and mutant p53 on stem cell survival and plasticity (Sabapathy).

A major requirement for GOF by mutant p53 is a constant stabilization of the mutant p53 protein, unlike the temporal accumulation of wt p53. The review by Vijayakumaran et al. summarizes the differences and similarities in the regulation of wt and mutant p53. While both wt and mutant p53 are inherently labile proteins and accumulate in response to stress, only the mutant form remains stable. Intriguingly, wt and mutant p53 share many of their regulatory mechanisms. However, the loss of the key negative autoregulatory loops due to mutation in p53 result in the sustained accumulation of mutant p53 following stress conditions or exposure to oncogenic stress in cancer cells. This, together with a loss of specificity of additional E3 ligases toward mutant p53, provides an explanation for the accumulation of mutant p53. The additional complexity of p53 regulation, both wt and mutant p53, by microRNA (miRNA) is presented Vijayakumaran et al. This reveals the ways by which p53 can be deregulated in cancer but, at the same time, may define potential new therapeutic targets.

Understanding the mechanisms by which mutant p53 gains its oncogenic functions are the subject of intensive research. While most of wt p53 activities are mediated through the transcriptional activation of target genes, the apoptotic activity of p53 also involves transcriptional independent activities. Giorgi et al. discussed the cytoplasmic apoptotic activities of wt p53, and the loss of these activities by mutations in p53. To date, there is no evidence for a GOF of mutant p53 directly regulating these activities (Giorgi et al.). On the other hand, it was reported that mutant p53 proteins can aberrantly cooperate with known transcription factors by leading to disregulated gene expression. This results in increased proliferation, invasion, genomic instability, and chemoresistance. The interaction of mutant p53 with p53 family members p63 and p73 is key to some of its GOF. Ferraiuolo et al. review the intricate relationship between mutant p53 and p63 or p73. Mutant p53 proteins can also hamper tumor suppression transcriptional programs by binding to and displacing the p53 family members p73 and p63 from their consensus of target gene promoters (4). Collectively, the intra-p53 family protein complexes with their oncogenic activity may represent druggable targets, which hold therapeutic potential.

Beyond p53 family members, Haupt et al. reviewed the major tumor suppressive pathways, which are subverted by mutant p53, including PTEN, PLK2, and PML, which control the cell cycle and the latter also the circadian clock. Intriguingly, mutant p53 deregulates cellular metabolism including glucose, lipid, and nucleotide metabolism, ensuring the sufficient supply of building blocks to support tumor growth (Haupt et al.). How are these plethora of activities achieved by mutant p53? At least two major mechanisms have been reviewed in this series. First, is by controlling gene expression through the alteration of specificity of certain transcription factors. Second, is by affecting chromatin remodeling through SWI/SNF and MLLs/MOZ (Haupt et al.). The effect of mutant p53 on MLLs/MOZ is achieved through ETS2, as reviewed in detail by Martinez. He discusses the mechanism by which mutant p53 protects ETS2 from degradation, and how this, in turn, affects the overall transcriptional effects of the ETS family and contributes to the oncogenic phenotype of mutant p53, such as increased nucleotide metabolic genes (Martinez). Bruno et al. reviewed the relationship of p53 with the cofactor Che-1/AATF. This provides an interesting example of a factor that acts as an activator and protector of both wt and mutant p53. In response to DNA damage, Che-1 induces the expression of wt and mutant p53, but activates wt p53 to induce growth arrest genes. In the case of mutant p53, it induces its expression and consequently the sequestration of p73 from apoptotic target genes, hence promotes survival (Bruno et al.).

A major consequence of mutant p53 GOFs is the acquired dependence of cancer cells on the expression of mutant p53. This dependence, which has been termed oncogenic addiction to mutant p53 has been discussed by multiple contributors to this series, highlighting its importance. Evidence for this addiction has been discussed by the Lozano, Sabapathy, and Del Sal groups. This addiction defines an Achilles Heal with important clinical implications. Parrales and Iwakuma highlighted the potential exploitation of heterozygosity, during which mutant p53 acts as a DN over wt p53, hence targeting mutant p53 eliminates its oncogenic driver and concurrently restores the tumor suppressive capacity of wt p53. Parrales and Iwakuma provided a comprehensive review of mutant p53 as a druggable target. They discuss the different classes of mutant p53 drugs, including compounds that restore wt p53 activity in cells expressing mutant p53, with the leading drug APR-246 (see below); compounds that deplete mutant p53 expression, where HSP90, in particular Ganetespib, is the most advanced drug currently in phase III clinical trial; and explore other approaches, which are currently used for other oncogenes, including knockdown and read-through of premature termination (Parrales and Iwakuma). This review was complemented by two focused reviews on mutant p53 therapeutics. The first by Bykov et al., which focused on the mechanism of action by APR-246, including the refolding of mutant p53, the impact on mutant isoforms of p53 family members p63 and p73 and the effect on the cellular redox regulators, primarily glutathione and thioredoxin, to enhance oxidative stress. The potential of APR-246 as a single agent and in combination with DNA damaging agents is discussed, and the current clinical status of APR-246 and prospects are outlined (Bykov et al.). The second therapeutic review by Burgess et al. focused on MDM2/MDMX targeted therapies. This review provides a thorough overview of the current drugs and approaches to target p53 *via* MDM2 and MDMX pathways. They outline the clinical development of current MDM2 targeting compounds. Importantly, they discuss the major hurdle in this approach, which is severe cytopenias. Although, this approach has not been designed to target mutant p53, the relevance of mutant p53 to this therapeutic approach and the availability of appropriate biomarkers were discussed (Burgess et al.).

## Concluding Remarks

Overall, this series of reviews on mutant p53 expose the pivotal role of mutant p53 as an oncogenic driver and outline the fast advancement in our understanding of its regulation and oncogenic activities. Our deeper understanding of mutant p53 also highlights clear limitations, such as the differences between mutants p53 proteins and between mouse and human mutant p53. The lack of appropriate mouse models for somatic p53 mutations, which represents the most common event in human cancer is a major hurdle to our understanding of mutant p53 to the cancer cell versus the microenvironment. The series also reflects the excitement around the clinical opportunities and current clinical development but highlights the need of potent molecules to specifically target mutant p53, given its prevalence in human cancers. It has also been increasingly clear that mutant p53 proteins are not a single entity, but they behave as a family of oncoproteins whose deciphering and therapeutic tackling might impact enormously on the success of threatening the vast majority of human cancers.

## Author Contributions

All authors listed, have made substantial, direct and intellectual contribution to the work, and approved it for publication.

## Conflict of Interest Statement

The authors declare that the research was conducted in the absence of any commercial or financial relationships that could be construed as a potential conflict of interest.
